# Differences in Executive Functioning Performance and Cortical Activation Between Autistic and Non-Autistic Youth During an fNIRS Flanker Task: A Pilot Study

**DOI:** 10.3390/brainsci16010065

**Published:** 2025-12-31

**Authors:** Jung-Mei Tsai, Jacob Corey, Daisuke Tsuzuki, Anjana Bhat

**Affiliations:** 1 Interdisciplinary Neuroscience Graduate Program, University of Delaware, 540 S College Avenue, Newark, DE 19713, USA; jungmei@udel.edu; 2 Biomechanics & Movement Science Program, Physical Therapy Department, University of Delaware, 540 S College Avenue, Newark, DE 19713, USA; coreyj@arcadia.edu; 3 Department of Physical Therapy, Arcadia University, 450 S Easton Rd, Glenside, PA 19038, USA; 4 Department of Information Science, Faculty of Science and Technology, Kochi University, Kochi 780-8520, Japan; tsuzukid@is.kochi-u.ac.jp; 5 Department of Psychological and Brain Sciences, University of Delaware, 540 S College Avenue, Newark, DE 19716, USA

**Keywords:** autism spectrum disorder, executive function, inhibitory control, functional near-infrared spectroscopy (fNIRS), Flanker task, cortical activation, neural correlates

## Abstract

**Background/Objectives**: Autism spectrum disorder is associated with executive functioning (EF) challenges, yet the neural correlates of EF challenges in autistic youth remain unclear. This study aimed to examine EF performance and cortical activation in autistic versus non-autistic youth, using functional near-infrared spectroscopy (fNIRS) during a modified Flanker task. **Methods**: Thirty age-matched (11.6 ± 0.8 years) autistic (N = 15) and non-autistic youth (N = 15) completed congruent and incongruent conditions of a modified Flanker task while cortical activation in prefrontal, parietal, and temporal regions was recorded using fNIRS. The Behavior Rating Inventory of Executive Function (BRIEF) was used to assess general EF impairments. Behavioral data (i.e., Flanker task mean reaction time/accuracy, and reaction time variability) and cortical activation were analyzed using ANCOVAs. Pearson correlations were used to determine the relationship between cortical activation, EF performance, and clinical measures. The significance level was set at *p* < 0.05, with FDR corrections for multiple comparisons. **Results**: While mean reaction time and accuracy were comparable across groups, autistic youth exhibited greater reaction time variability (autistic youth = 34.8 ± 10.36; controls = 26.4 ± 1.94, *p* = 0.02, Hedges’ *g* = 0.85) and higher BRIEF index scores compared to controls (*p*_s_ < 0.001, Hedges’ *g*s > 1.3; e.g., Global Executive Composite Score for autistic youth = 71.3 ± 3.7; controls = 47.8 ± 2.4), indicative of delayed EF development. During the incongruent condition, compared to non-autistic controls, autistic youth showed lower left inferior parietal lobe (IPL) activation (Mean HbO_2_ in autistic youth = −0.02 ± 0.006 mmol.mm; controls = 0.01 ± 0.006 mmol.mm, *p*_s_ < 0.001, Hedges’ *g* = 0.5) and a lack of left-lateralized activation (e.g., left vs. right STS activation, *p* < 0.001, Hedges’ *g* = 0.41 in the non-autistic youth). In the ASD group, lower activation in the left STS was associated with lower EF performance (r = −0.28, *p* = 0.007), whereas greater activation in various right-hemispheric ROIs was associated with better EF performance (r = −0.31 to −0.35, *p*_s_ < 0.005), suggesting potential compensatory activation. **Conclusions**: The findings revealed ASD-specific differences in the neural correlates of EF performance and possible alternative compensatory activation patterns. These potential neural correlates of EF performance highlight the utility of fNIRS-based neural measures to better understand the neural bases of EF differences in autism. **Study Registration**: This study was approved by the Institutional Review Board (IRB) at the University of Delaware (Protocol #: 1947455) on 4 October 2022.

## 1. Introduction

Autism spectrum disorder (ASD) is characterized by core symptoms of social communication difficulties, including delays in verbal and nonverbal communication, as well as the presence of repetitive behaviors or restricted interests [1]. Apart from these core symptoms, autistic individuals also face co-occurring challenges in executive functioning (EF) [2], including poor cognitive flexibility [3,4], impaired working memory [2], and difficulties with inhibitory control [5,6]. The study of EF challenges in autistic individuals is of particular importance due to their prevalence and association with other skills/traits such as social functioning, sensory processing, daily living skills, repetitive behaviors, and motor skills [7,8,9,10,11]. The present study aims to examine differences in cortical activation between autistic and non-autistic youth during a specific EF component, known as inhibitory control (IC), using a modified Flanker task involving two conditions: congruent and incongruent, and concurrent use of functional near-infrared spectroscopy (fNIRS).

### 1.1. Co-Occurring Difficulties in Inhibitory Control in Autistic Individuals

EF is an overarching cognitive skill responsible for top-down regulation of social interaction, communication, and daily living skills. It comprises a variety of constructs, including inhibitory control, cognitive flexibility, and working memory [5,12]. Inhibitory control (IC), also known as cognitive control, is the ability to suppress undesired, irrelevant, or inappropriate responses by engaging in interference control and response inhibition [2]. Interference control involves ignoring irrelevant information while processing target stimuli, whereas response inhibition refers to the suppression or cancelation of dominant or habitual motor responses [3,11,13]. Together, these components enable IC, which is foundational for effective communication, social interaction, and daily living skills. Autistic individuals experience greater IC challenges compared to neurotypical individuals, which considerably impact their social communication and repetitive behaviors [5,6,14]. For instance, individuals with IC difficulties often struggle to understand conversations, resulting in literal interpretation of words and phrases, a common challenge for autistic individuals [5]. Additionally, response inhibition is linked to higher-order repetitive behaviors, including fixated interests and ritualistic behaviors [15]. Overall, IC is closely related to the core symptoms of ASD, including social communication delays and repetitive behaviors; therefore, it is important to understand EF or IC differences in autistic youth compared to non-autistic youth.

### 1.2. The Neural Mechanisms Underpinning EF Challenges in Autistic Individuals

While considerable research has focused on the neural correlates of core ASD symptoms, the neural correlates of domain-specific EF challenges in autistic youth are less understood [16,17]. In terms of the neural correlates of EF challenges, some suggest that it may arise from differential activation in the Default Mode Network (DMN) and Central Executive Network (CEN) regions, along with their hypo- or hyperconnectivity with other networks. The DMN is activated during introspective thought, creative thinking, and imagination, but is suppressed during goal-directed tasks that require EF [18,19,20]. The DMN includes cortical regions of the superior temporal sulcus (STS) between the superior and middle temporal gyri; regions within the inferior parietal lobe (IPL), such as supramarginal and angular gyrus; subdivisions of the prefrontal cortex (PFC), such as dorsomedial and ventromedial PFC and the superior and middle frontal gyrus (SFG and MFG); and other areas, including the retrosplenial cortex and the anterior/posterior cingulate cortices. In contrast, the CEN is activated when processing external stimuli and performing goal-directed tasks that require EF. The CEN comprises the dorsolateral PFC, supplementary motor area (SMA), MFG, IPL, and posterior parietal cortices [20,21]. Together, these two networks, along with their overlapping regions, play a vital role in daily EF performance. Failure to suppress specific DMN regions during EF tasks or alterations in activation within the two networks may lead to EF challenges. A meta-analysis of fMRI studies in autistic and neurotypical individuals during EF tasks found ASD-related differences in functional activation, i.e., autistic individuals exhibit atypical activation in the left and right medial frontal gyrus and other prefrontal and subcortical regions during EF tasks compared to neurotypical individuals [22].

### 1.3. Autism-Related Differential Neural Activity Involved in IC

Several fMRI studies and some fNIRS studies have investigated the neural correlates of IC in autistic individuals with typical cognition and fewer support needs. Overall, meta-analyses of previous studies indicate that specific brain areas, including frontal cortices (MFG and IFG), IPL, and STS, are associated with IC challenges in autistic individuals [23,24]. Yet, few fMRI studies have assessed neural correlates during Flanker tasks in autistic individuals, and existing studies typically involve small samples and individuals with typical cognitive abilities [25,26,27]. On the other hand, fNIRS offers the advantage of engaging in naturalistic interactions in seated positions while tolerating motion artifacts, making it an ideal choice for autistic children who are unable to remain still in an fMRI scanner. A meta-analysis of fNIRS studies examining prefrontal cortical activation during IC tasks, such as Stroop and Go/No-go tasks, found that autistic adolescents showed lower PFC activation compared to neurotypical controls, which was associated with their IC performance [28]. Interestingly, the study found that autistic adolescents showed greater PFC activation during working memory tasks compared to the controls, underscoring the value of testing neural correlates using different EF tasks [28].

However, to our knowledge, no studies have administered an fNIRS Flanker task with non-social stimuli to compare cortical activation between autistic children and non-autistic controls. Only one study by Chen et al. [29] presented an fNIRS Flanker task using facial expressions as stimuli, rather than the non-social stimuli shown in typical IC tasks, and found that autistic adolescents did not exhibit faster reaction times during congruent conditions (i.e., where the target stimulus was identical to the distractor stimuli) versus incongruent conditions (i.e., where the target stimulus conflicted with the distractor stimuli), a phenomenon commonly observed in non-autistic individuals during IC tasks. Autistic children showed greater right- vs. left-hemispheric PCG activation when inhibiting emotional stimuli [29]. Taken together, the neural correlates of IC challenges in autistic individuals may be associated with hypo- or hyperactivation in DMN and CEN regions. However, more studies and different tasks, such as the Flanker task, are still lacking to examine the neural correlates of IC in autistic children who need more support. Therefore, the present study aimed to compare IC performance and cortical activation between 15 autistic youth and 15 age-matched non-autistic controls during an fNIRS Flanker task involving congruent and incongruent conditions. We hypothesized that, (1) during the fNIRS task, autistic youth would exhibit worse IC performance (i.e., slower mean reaction times, greater reaction time variability, and lower mean accuracy) compared to non-autistic youth, and (2) cortical activation patterns would differ between autistic and non-autistic youth in DMN- and CEN-related cortical regions.

## 2. Materials and Methods

### 2.1. Participants

Thirty autistic and non-autistic youth between the ages of 5 and 16 participated (11.46 ± 0.85 years of age, nine males and six females for non-autistic youth; 11.59 ± 0.81 years of age, twelve males and three females for autistic youth). We recruited 15 autistic youth prior to recruiting non-autistic youth (NASD group). We then recruited youth for the NASD group to match the ASD group by age. Sample size estimates for detecting group differences were based on samples reported in our prior fNIRS publications comparing cortical activation between autistic and non-autistic youth during other cognitive/motor response tasks [30,31]. Youth were recruited through online postings, flyers, and the Simons Powering Autism Research (SPARK) participant research match service throughout the study period (https://www.sfari.org/resource/spark/). Prior to participation, families completed a screening and an interview to confirm their eligibility. Inclusion criteria for the ASD group were as follows: (a) the participant could follow two-step directions; and (b)the participant had an ASD diagnosis confirmed by a school psychologist and/or an Individualized Education Plan (IEP) for ASD-related services and/or a medical/neuropsychological record from a psychiatrist or clinical psychologist using the Autism Diagnostic Observation Schedule (ADOS) [32] and/or the Autism Diagnostic Interview–Revised (ADI-R) [33]. Participants in both groups were excluded if they had any behavioral or sensory issues that prevented them from completing the test activities. NASDs were excluded if they had a family history of ASD.

Parents in both groups completed additional standard screening questionnaires shown in Table 1. The Social Responsiveness Scale–Second Edition (SRS-2) [34] measures social responsiveness impairments, with a T-score of 60–75 indicating mild-to-moderate impairment and a T-score above 75 indicating severe impairment. The Coren Handedness Questionnaire [35] assessed handedness, with 27 out of 30 participants being right-hand dominant.

The Vineland Adaptive Behavior Scale–Third Edition (VABS-3) [36] measures adaptive functioning based on standard scores for communication, socialization, daily living, and motor domains. The first three domains generate an adaptive behavioral composite (ABC) score that represents overall adaptive functioning, according to which all non-autistic children had adequate functioning, two autistic children had adequate functioning, and ten had moderately low functioning.

The Behavior Rating Inventory of Executive Function (BRIEF) [37] measured baseline EF impairments, with a T-score of 60–64 indicating mildly elevated impairments and 65 or above indicating clinically elevated EF impairments. The BRIEF contains sixty-three items across eight domains of EF: Inhibit, Shift, Emotional Control, Initiate, Working Memory, Plan/Organize, Organization of Materials, and Monitor. These eight domains produced eight domain-specific T-scores, % scores, and three index T-scores (Table 1).

Finally, the Bruininks–Oseretsky Test of Motor Proficiency, Second Edition (BOT-2) [38] assessed motor performance, in which the manual coordination and body coordination subscales were used to measure fine and gross motor coordination performance. The subscale resulted in standard scores, with higher scores indicating better performance.

The two groups did not differ on any demographic measures such as race, ethnicity, sex, age, or handedness. However, compared to non-autistic youth, autistic youth had lower VABS-3, BOT-2 body coordination, and manual coordination scores, and higher SRS-2 total scores and BRIEF index scores, indicating challenges in adaptive functioning, communication, and body/manual coordination, as well as elevated risk of motor, social, and EF impairments (*p*_s_ < 0.05, adjusted for multiple comparisons) (Table 1). Upon enrollment, written informed consent and written assent were obtained from the participating youth and their caregivers. Verbal assent was obtained only when written assent was rendered inappropriate due to limited reading comprehension. All procedures adhered to the principles of the Declaration of Helsinki, and the study protocol was approved by the University of Delaware Institutional Review Board (UD IRB, Study Approval #: 1947455). Data from the screening and additional questionnaires were collected and managed using REDCap electronic database hosted at the University of Delaware [39,40].

### 2.2. Experimental Procedures

**Figure 1:**
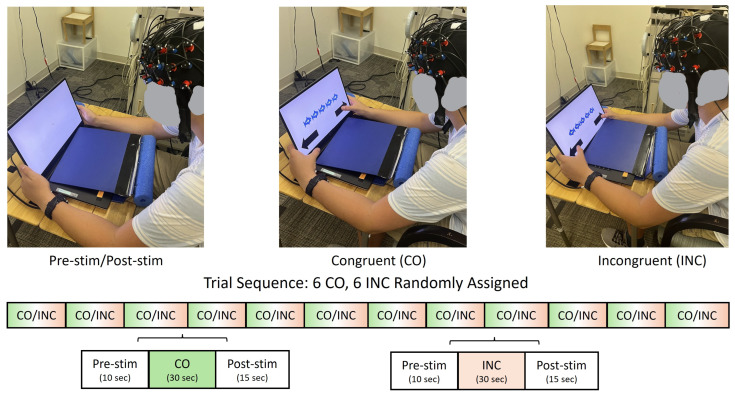
NIRS Flanker task. The task involved twelve trials (six per condition)—congruent (CO) or incongruent (INC), using a randomized block design. Each trial had a 10 s pre-stimulation period, a 30 s stimulation period, and a 15 s post-stimulation period. Participants were instructed to press the arrow pointing in the direction that the center fish was looking.

**Figure 2:**
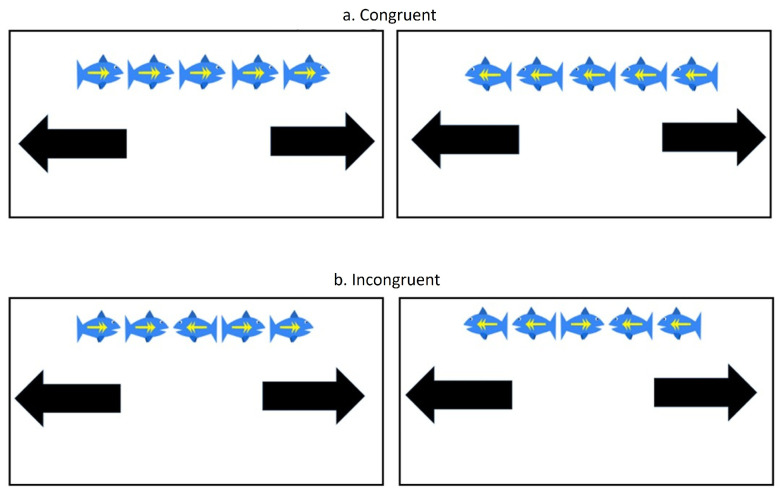
Stimuli during the congruent and incongruent conditions of the modified Eriksen Flanker task. (**a**) During the congruent condition, all five fish stimuli faced the same direction. (**b**) During the incongruent condition, the center fish faced the opposite direction to the side fish. Participants were instructed to press the arrow pointing in the direction that the center fish was looking.

During the fNIRS experiment, the child was seated across a table wearing an fNIRS cap that held a 3 × 11 fNIRS probe configuration (Figure 1). To assess IC performance, each child completed the modified Eriksen Flanker Task programmed in E-Prime 3.0 Software (version 3.0.3.219), with a touchscreen laptop display (Psychology Software Tools, Inc., Pittsburgh, PA, USA, Figure 1) [41]. The sampling rate of the touchscreen display was 125 Hz, and the E-prime trigger’s timing accuracy was <0.5 milliseconds. The task consisted of 12 trials, totaling 11 min, with 6 trials per condition (i.e., congruent or incongruent) presented in random order. Within each trial, a series of fish stimuli were presented as follows: (a) during congruent conditions: five fish were facing in the same direction (e.g., “
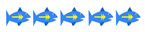
” or “
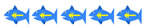
”); and (b) during incongruent conditions, the outer four fish faced the same direction while the center fish faced the opposite direction (e.g.,“
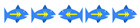
” or “
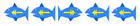
”) (Figure 2). The stimulus counts per trial varied from child to child based on the child’s response speed, with the inter-stimulus interval (ISI) being 0 to 3 s. Note that multiple responses were recorded per trial and averaged to represent each trial. However, we had a small sample (N = 15 per group) and six trials or data per condition per participant were obtained using a randomized block design; hence, to improve statistical power, trial-level analysis (vs. subject-level analysis) was conducted, as the research question focused on how performance and activation differed between the two Flanker task conditions and between the two groups (ASD vs. NASD).

Participants were instructed to click the arrow at the bottom of the screen to indicate the direction of the center fish and to respond as quickly and accurately as possible (Figure 1 and Figure 2). Each trial began with a 10 s pre-stimulation baseline, followed by a 30 s stimulation period (i.e., fish presentations) and a 15 s post-stimulation baseline period. During the baseline period (10 s pre-stimulation and 15 s post-stimulation), the participant was asked to attend to the crosshair on the screen. If the participant did not respond during the stimulus period, the subsequent trial automatically began 3 s later; if the participant responded within the stimulus period, the time from stimulus presentation to the arrow press was recorded as the reaction time. The experiment was terminated after 12 trials were completed. We recorded the experimental procedure using two synchronized camcorders that recorded the child’s upper body view, screen display, and finger responses to ensure task accuracy and compliance (Figure 2).

### 2.3. FNIRS Data Collection

**Figure 3:**
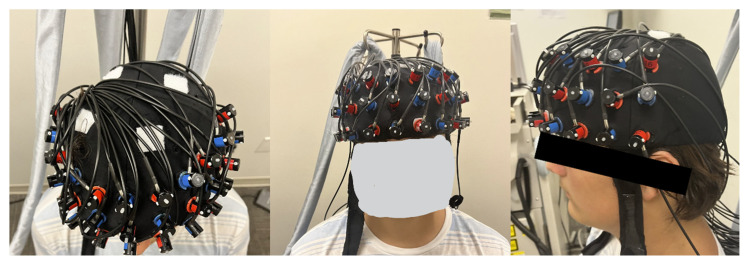
The 52-channel fNIRS setup.

We used the Hitachi fNIRS system (ETG-4000, Hitachi Medical System, Inc., Tokyo, Japan) to measure relative hemodynamic changes in the various regions of interest (ROIs). The 3 × 11 probe sets consisted of 17 infrared emitters and 16 detectors with a fixed inter-probe distance of 30 mm (Figure 3). The probe array included a total of 52 channels. The emitters emitted infrared light of two wavelengths (695 and 830 nm) at 10 Hz, which passed through the skull and reached the cortical regions just below the probes. The attenuation of infrared light was used to calculate changes in hemoglobin concentration (∆Hb) of oxygenated (∆HbO_2_) and deoxygenated hemoglobin (∆HHb) per channel using the modified Beer–Lambert law [42]. The cap was placed over bilateral frontal, parietal, and temporal regions, with the midline aligned with the tragus and the probe’s bottom row aligned with T3 of the International 10–20 system. We used the E-Prime presentation software (version 3.0) to present the stimuli on the screen, and that also triggered the Hitachi fNIRS system (Model: ETG-4000) to ensure synchrony between E-Prime and the Hitachi system. All fNIRS experiments were video-recorded using two synchronized camcorders.

### 2.4. Spatial Registration and ROI Assignment

**Figure 4:**
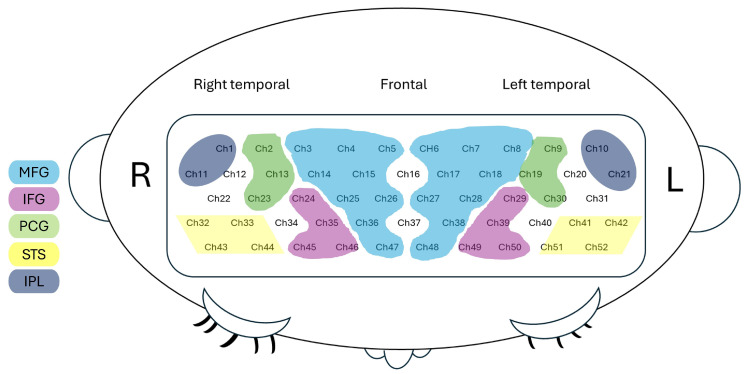
ROI assignments for 44 of the 52 channels.

Using a Polhemus motion analysis system, we registered the 3D position of each fNIRS probe and standard cranial landmarks (nasion, inion, left and right tragus) to a reference coordinate system. The 3D coordinates on the reference coordinate system of each channel were then transformed to the Montreal Neurological Institute (MNI)’s coordinate system using spatial registration methods developed by Tsuzuki et al. [43]. Using the MNI coordinates, we then estimated the channel positions within a standardized 3D brain atlas based on structural information from an anatomical database of 17 adults [44]. The estimated channel locations were labeled using the LONI Probabilistic Brain Atlas (LPBA), based on MRI scans of 40 healthy adults [45]. Each channel was considered the centroid of a sphere. We assigned each channel to a specific ROI if the given ROI covered 60% of the channel area. Forty-four out of fifty-two channels were assigned to specific ROIs. The assigned ROIs are illustrated in Figure 4 and listed below. The MFG ROI was represented by channels 3, 4, 5, 14,15, 25, 26, 18, 36, and 47 on the right and channels 6,7, 8, 17, 18, 27, 28, 38, and 48 on the left. The IFG ROI was represented by channels 24, 35, 45, and 46 on the right and channels 29, 39, 49, and 50 on the left. The PCG ROI was represented by channels 2, 13, and 23 on the right and channels 9, 19, and 30 on the left. The STS ROI was represented by channels 32, 33, 43, and 44 on the right, and channels 41, 42, 51, and 52 on the left. Finally, the IPL ROI was represented by channels 1 and 11 on the right and channels 10 and 21 on the left. Specific channels on the right (channels 12, 22, and 34) and left (channels 20, 31, and 40) were excluded from ROI assignments due to spatial ambiguity, either when one of the two homologous channels fell within different ROIs or when a channel did not cover 60% or more of a given ROI. The detailed ROI assignments are listed in Supplementary Table S1.

### 2.5. Data Processing

We utilized custom MATLAB code for data processing, which incorporated functions from the open-source software Hitachi PoTATo, version 3.9 [46] and Homer, version 2 [47]. As reported in previous publications [30,31], data from each channel were first band-pass filtered between 0.01 and 0.5 Hz to remove low-/high-frequencies related to respiratory and cardiovascular signals. We then applied the wavelet method to remove movement artifacts and used General Linear Modeling (GLM) to estimate the hemodynamic response function [46,47]. To correct baseline drifts, we subtracted the linear trend between the pre-stimulation and post-stimulation baselines from values in the stimulation period using a Hitachi PoTATo function [46]. Data were plotted and visually inspected at each step of the analysis to exclude channels/trials with poor contact or persistent motion artifacts (i.e., sudden spikes with amplitudes over 1, flat lining, etc.). The total data retention rates for the NASD and ASD groups were 93.3% and 97.9%, respectively. Data retention rates (%) per channel, group, and condition are reported in Supplementary Tables S2 and S3. HbO_2_ data were then averaged across channels for each ROI. Subsequently, outlier analysis was conducted for each ROI within each condition and group to ensure all data were within two standard deviations of the group averages. Cortical activation was defined as the baseline-corrected average HbO_2_ value during the stimulation period of a given trial.

### 2.6. Behavioral Data Coding

Video recordings of the fNIRS tasks were screened by a student coder and the first author to determine task compliance based on finger and whole-body movements. A given trial was excluded from analysis if a participant failed to follow instructions or exhibited challenging behaviors leading to movement artifacts.

### 2.7. Statistical Analysis

To examine the cortical activation differences between the NASD and ASD groups, we conducted ANCOVAs using average cortical activation (HbO_2_) values using the within-group factors of Condition (congruent, incongruent), Hemisphere (left, right), and Region (MFG, IFG, PCG, IPL, STS), and a between-group factor of Group (ASD, NASD). Due to the wide age range, we used Age as a covariate in the analysis. Similarly, to examine behavioral effects, we conducted ANCOVAs on behavioral performance during the Flanker task (i.e., mean reaction time, reaction time variability, and mean accuracy) using Condition as a within-group factor, Group as a between-group factor, and Age as a covariate. Post hoc comparisons were performed when Condition × Group and related interaction effects were significant. We used independent *t*-tests to compare cortical activation between groups and paired *t*-tests to compare condition-related differences within each group. Reaction time variability was calculated as the coefficient of variation (CV) of reaction time. The sphericity assumption was tested using Mauchly’s test of sphericity, and if the sphericity assumption was violated, Greenhouse–Geisser corrections were applied (Greenhouse–Geisser ε values = 0.745–0.838).

To investigate the relationship between cortical activation, functioning, and task performance, Pearson correlations were used to correlate cortical activation with mean reaction time and reaction time variability during the modified Flanker task, as well as parent-reported clinical measures of adaptive functioning using the VABS-3, motor performance using the BOT-2 (manual coordination), cognitive performance using the BRIEF (index scores), and autism severity using the total SRS-2 score. The significance level was set at *p* < 0.05. We applied the false discovery rate (FDR) correction to the significance criteria when multiple comparisons were made. We reported effect sizes using partial eta-squared (*η*^2^*_p_*) for the ANCOVAs, with values of 0.01 considered small, 0.06 medium, and 0.14 large. Hedges’*g*s were calculated to measure the effect sizes for post hoc *t*-tests, which were interpreted as “small” (Hedges’ *g* < 0.5), “medium” (Hedges’ *g* > 0.5), and “large” (Hedges’ *g* > 0.8), similar to Cohen’s d benchmarks.

## 3. Results

### 3.1. Behavioral Differences

**Figure 5:**
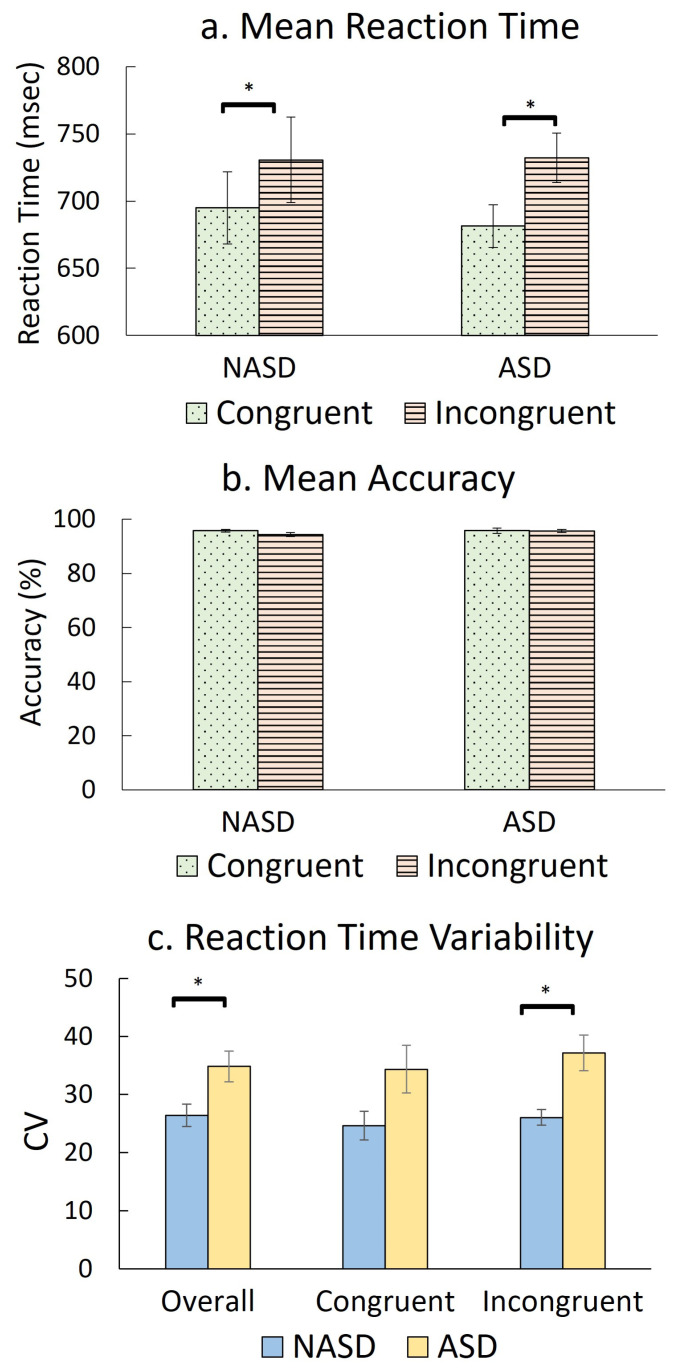
Condition-related effect on accuracy (**a**) and reaction time (**b**), and group effect on reaction time variability (**c**) during the Flanker task. (**a**) Both autistic (ASD) and non-autistic (NASD) youth responded more slowly to incongruent stimuli compared to congruent stimuli (* *p*_s_ = 0.001–0.022). (**b**) No main or interaction effects of Group or Condition were found for mean accuracy. (**c**) Autistic youth had significantly greater reaction time variability compared to non-autistic youth in both conditions (* *p*_overall_ = 0.017, *p*_incongruent_ = 0.004), indicating lower response consistency in autistic youth.

To examine changes in behavioral variables, we conducted repeated-measures ANCOVAs on mean reaction time, reaction time variability, and mean accuracy, using the within-group factor of Condition, the between-group factor of Group, and Age as a covariate (see Supplementary Table S10 for statistical details). In terms of mean reaction time, the ANCOVA revealed main effects of Condition (F(1,177) = 6.58, *p* = 0.011, *η*^2^*_p_* = 0.04) and Age (F(1,177) = 191.78, *p* < 0.001, *η*^2^*_p_* = 0.52), indicating that when data from both groups were combined, mean reaction times differed between the two conditions and across age groups. The condition effect of interest did not covary with age; thus, to examine the condition-related effects, post hoc *t*-tests were conducted between conditions for both groups (see Figure 5a). There were no main effects of Group or a Group x Condition interaction, indicating that mean reaction times did not differ between autistic and non-autistic youth regardless of condition; hence, group differences were not further examined. In terms of condition-related effects on mean reaction time, within-group *t*-tests revealed that both groups were slower during the incongruent condition compared to the congruent condition (*p*_s_ = 0.001–0.022, *g* = 0.13–0.31, see Figure 5a and Supplementary Table S4), indicating a greater cognitive load imposed by the incongruent condition. For mean accuracy, there were no significant main or interaction effects to test further (see Figure 5b).

In terms of reaction time variability, only a main effect of Group was found (F(1,177) = 51.02, *p* < 0.01, *η*^2^*_p_* < 0.001), indicating that reaction time variability differed between ASD and NASD groups regardless of condition; therefore, the data were pooled to explore the group effect on reaction time variability (Figure 5c), which revealed that the ASD group exhibited significantly greater reaction time variability compared to the NASD group across both conditions (*p* = 0.017, *g* = 0.85, see Figure 5c and Supplementary Table S5). In terms of reaction time variability, there were no other significant main or interaction effects (i.e., Condition or Condition x Group), suggesting both groups showed a consistent pattern across conditions. Overall, only reaction time variability differed between groups, indicating lower response consistency in the ASD group compared to the NASD group.

### 3.2. Differences in Cortical Activation

**Figure 6:**
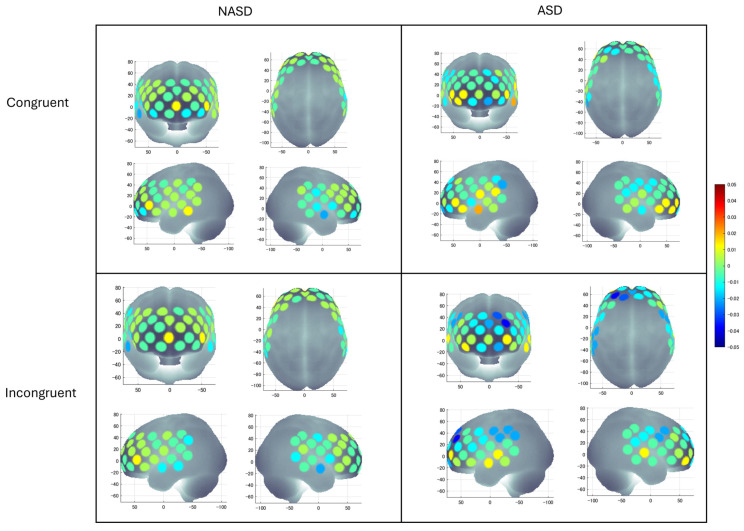
A visual representation of average HbO_2_ concentration during the congruent and incongruent conditions in autistic (ASD) and non-autistic (NASD) youth. HbO_2_ values on the Y-axis range from −0.05 (blue) to 0.176 (red). This figure was made in MATLAB (The MathWorks Inc. (2021). version 2021b, The MathWorks, Inc., Natick, MA, USA, https://www.mathworks.com).

ANCOVA on HbO_2_ concentration revealed main effects of Hemisphere (F(1,177) = 7.90, *p* = 0.006, *η*^2^*_p_* = 0.043), Region (F(3.051,176) = 3.01, *p* = 0.029, *η*^2^*_p_* = 0.017), two-way interactions of Hemisphere × Group (F(1, 176) = 8.87, *p* = 0.003, *η*^2^*_p_* = 0.048), Region × Group (F(3.05, 177) = 3.38, *p* = 0.017, *η*^2^*_p_* = 0.019), Hemisphere × Region (F(2.98,177) = 4.29, *p* = 0.005, *η*^2^*_p_* = 0.024), three-way interaction of Hemisphere × Region × Group (F(2.98,177) = 4.75, *p* = 0.003, *η*^2^*_p_* = 0.026), and four-way interaction of Condition × Hemisphere × Region × Group (F(3.19,177) = 2.63, *p* = 0.046, *η*^2^*_p_* = 0.015) (Supplementary Table S9 for detailed statistics). No other significant main or interaction effects were found. The significant four-way interaction indicated that cortical activation patterns across hemispheres and ROIs differed between groups as a function of Condition. The four-way interaction did not covary with Age and was therefore further examined through post hoc *t*-tests. The visual representation of the average HbO_2_ concentration during both conditions in both groups is shown in Figure 6. The means and standard errors (SEs) of HbO_2_ concentrations are shown in Supplementary Table S6, and the *p*-values of post hoc comparisons, direction of effects, and effect sizes are shown in Supplementary Table S7. It is important to note that no significant condition-related differences were observed in either group, and other significant main/interaction effects were not of interest; therefore, those differences are not discussed here.

### 3.3. Group Differences in Cortical Activation

**Figure 7:**
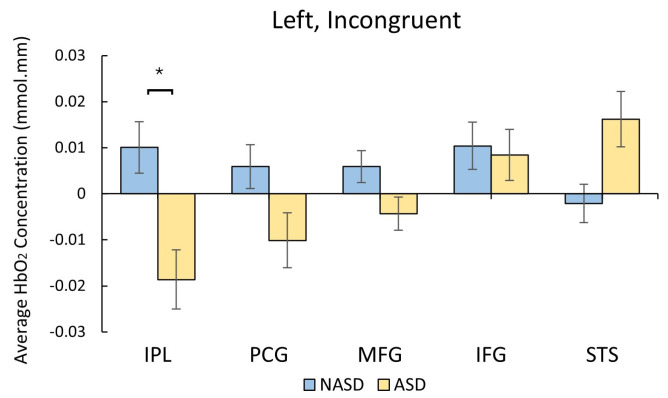
Group differences in cortical activation during the fNIRS Flanker task. Autistic youth (ASD) showed lower left IPL activation during the incongruent condition compared to non-autistic youth (NASD) (* *p* < 0.001, significant post-FDR corrections).

During the incongruent condition, compared to the NASD group, the ASD group exhibited lower left IPL activation (*p* < 0.001, Hedges’ *g* = 0.5, Figure 7, Supplementary Tables S6 and S7).

### 3.4. Hemispheric Differences in Cortical Activation

**Figure 8:**
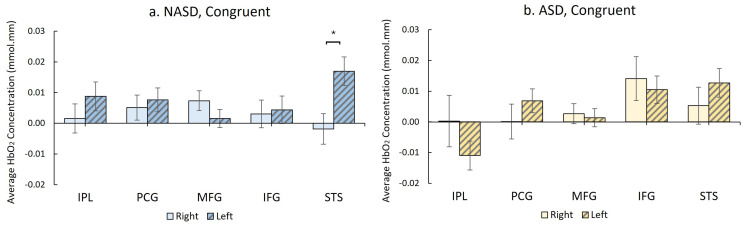
Hemispheric differences in regional activation during the fNIRS Flanker task. (**a**) Non-autistic youth (NASD, shown in blue) exhibited greater left than right STS activation during the congruent condition (* *p* < 0.001, survived FDR correction), whereas (**b**) autistic youth (ASD, shown in yellow) did not exhibit such lateralized activation.

During the congruent condition, the NASD group showed greater left than right STS activation, i.e., more left-lateralized activation (*p* < 0.001, g = 0.412, Figure 8a, Supplementary Tables S6 and S7). In contrast, the ASD group did not show significant hemispheric differences in activation in all ROIs during the congruent condition (Figure 8b, Supplementary Tables S6 and S7).

### 3.5. Regional Differences in Activation

**Figure 9:**
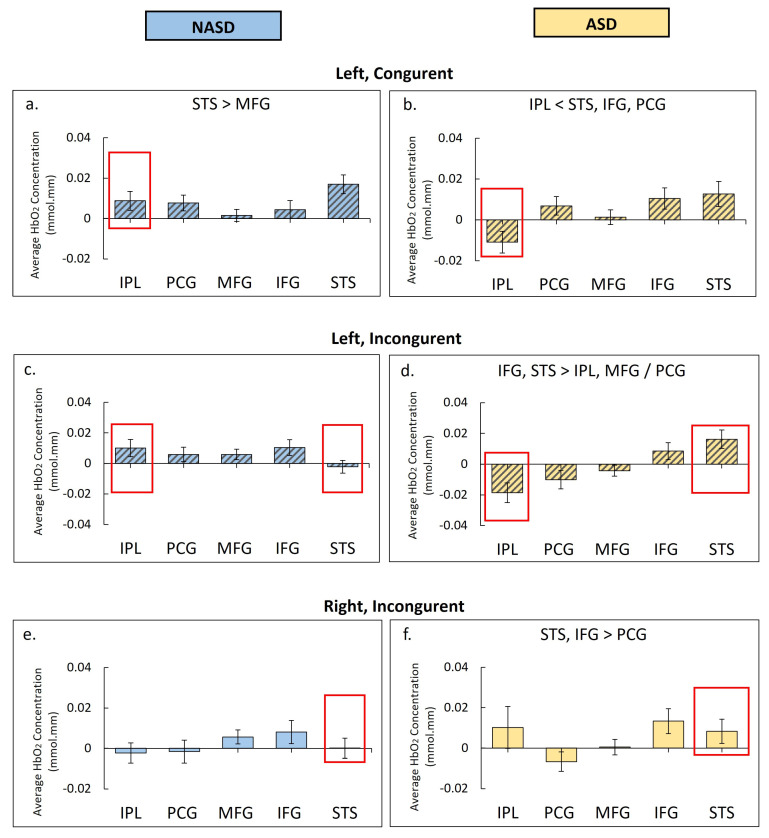
Regional differences in fNIRS cortical activation in non-autistic youth (NASD, shown in blue) and autistic youth (ASD, shown in yellow). During the congruent condition, the ASD group (**b**) exhibited lower IPL activation in the left hemisphere (IPL < STS, IFG, and PCG. *p*_s_ < 0.01), whereas the NASD group (**a**) did not (IPL > STS, *p* = 0.006, see red boxes in congruent condition figures). For the congruent condition, there were no significant regional differences in right-hemispheric activation in both groups. During the incongruent condition, the ASD group (**d**) showed STS hyperactivation and lower IPL activation in the left hemisphere (IFG, STS > IPL, MFG/PCG, *p*_s_ < 0.005), whereas the NASD group (**c**) did not, see red boxes in incongruent condition figures. During the incongruent condition, the ASD group (**f**) showed hyperactivation of the STS ROI in the right hemisphere (STS, IFG > PCG, *p*_s_ = 0.003, 0.006), while the NASD group (**e**) did not. Only significant differences that survived FDR correction are listed.

During the congruent condition, within the left hemisphere, the NASD group showed greater STS than MFG activation (*p* = 0.006, g = 0.42, Figure 9a, Supplementary Tables S6 and S7), whereas the ASD group showed greater STS, IFG, and PCG activation compared to IPL activation (*p*_s_ < 0.01, g = 0.38–0.44, Figure 9b), with both groups showing the greatest activation in the STS, but only the ASD group showing the lowest activation in the IPL ROI. During the congruent condition, within the right hemisphere, no regional differences were seen in the NASD or ASD groups.

During the incongruent condition, within the left hemisphere, the NASD group showed no regional activation differences (Figure 9c), whereas the ASD group showed greater STS and IFG activation than IPL, MFG, or PCG activation (Figure 9d, *p*_s_ < 0.005). During the incongruent condition, within the right hemisphere, no regional differences were seen in the NASD group (Figure 9e), whereas the ASD group showed greater STS and IFG than PCG activation (*p*_s_ < 0.01, g = 0.29–0.39, Figure 9f). See Supplementary Tables S6 and S7 for average cortical activation and post hoc comparison details.

### 3.6. Correlating Cortical Activation, Behavioral Performance, and Clinical Measures in the ASD Group

In the ASD group, during the Flanker task’s congruent condition, autistic youth with faster reaction times had lower right IPL and IFG activation (r = 0.29–0.39, *p*_s_ < 0.01, Supplementary Table S8) and greater left STS activation (r = −0.28, *p* = 0.007), but those with more consistent responses (i.e., lower reaction time variability) had greater right IPL activation. During the incongruent condition, autistic children with faster reaction times had lower right PCG activation (r = 0.38, *p* < 0.001).

When correlating cortical activation with clinical measures, autistic children with better social responsiveness on the SRS-2 had greater right IPL activation during the congruent condition (r = −0.3, *p* = 0.004). Lastly, autistic youth with better EF based on BRIEF scores showed greater right IPL and IFG activation during the congruent condition (r = −0.31 to −0.35, *p*_s_ < 0.005), but lower left PCG activation during the incongruent condition (r = 0.35 to 0.38, *p* < 0.001, Supplementary Table S8). No other correlations were significant. Overall, in the ASD group, better EF performance, as seen by lower/consistent reaction times and better BRIEF scores, was associated with differential right IPL, IFG, and PCG and left STS cortical activation. Note that all significant correlations survived FDR correction.

## 4. Discussion

This preliminary study compared EF performance and cortical activation patterns between autistic and non-autistic youth during an fNIRS Flanker IC task. For behavioral performance, autistic youth had greater reaction time variability corresponding to higher BRIEF index scores, indicating delayed/impaired EF performance. For cortical activation, autistic youth exhibited differences between groups and hemispheres among regions corresponding to DMN and CEN. Moreover, cortical activation in the IPL, STS, IFG, and PCG regions was associated with Flanker task performance, SRS-2, and BRIEF scores. The following sections discuss these preliminary findings and how they may explain EF challenges in autistic youth.

### 4.1. Differences in Behavioral Performance

In this study, autistic youth exhibited greater reaction time variability compared to non-autistic youth during the fNIRS Flanker task. Across various cognitive, motor, and speech tasks, reduced behavioral variability during the task is considered an indicator of neural and developmental maturity [48,49,50]. Therefore, greater reaction time variability during the Flanker task may indicate that autistic children have delayed IC development compared to age-matched, non-autistic youth. They also showed significantly higher BRIEF indices compared to non-autistic youth, with large effect sizes, indicating an elevated risk of general EF impairments. These findings aligned with other meta-analyses reporting that, compared to neurotypical controls, autistic youth show small-to-medium differences in IC performance (n = 5140) when assessed using direct measures [6], but large differences using indirect measures such as parent reports (n = 985) [6].

In this study, autistic youth exhibited typical mean reaction times and accuracy during the Flanker task. Similarly, previous studies have reported no difference in EF performance as measured by Flanker task reaction time and accuracy compared to neurotypical controls (i.e., no Group × Condition effects) [51,52]. Another study reported no autism-related differences in mean reaction time or accuracy during the original Flanker task; however, autistic children had slower reaction times and lower accuracy compared to non-autistic controls when the distractor sizes were 20% to 40% smaller than the target stimulus [53]. Findings of typical mean reaction times and accuracy, alongside greater individual reaction time variability in autistic youth compared to non-autistic controls, align with past literature reporting delayed/variable, but not necessarily entirely atypical, EF performance in autistic individuals.

### 4.2. Differences in fNIRS-Based Cortical Activation

Past studies have reported differences in neural activation during EF tasks in autistic individuals compared to controls [15,54,55], suggesting that neural activation patterns may be a reliable indicator of IC differences in autistic individuals. The current study indicated that during the incongruent condition, autistic youth exhibited lower left IPL activation. Interestingly, in autistic youth, greater activation of right hemisphere ROIs (IPL and IFG) was associated with better EF performance as measured by baseline BRIEF scores, indicating a potential compensatory activation pattern in autistic youth.

The above ROIs coincide with networks critical for EF, including the DMN- and the CEN-related ROIs, and provide potential neural correlates of IC challenges in autistic youth. Together, these atypical patterns of activation, greater right IPL, IFG, and PCG activation in autistic youth, may suggest a potential compensatory mechanism leading to more variable yet intact EF performance.

Additionally, during the incongruent conditions, autistic youth showed trends of lower left PCG and left MFG activation compared to non-autistic youth. The MFG and PCG regions are considered important for IC due to their role in selective attention (i.e., directing attention to relevant stimuli while inhibiting attention to irrelevant stimuli) [56,57,58] and shifting attention [59]. In the present study, the pattern of lower PCG activation seen in autistic youth was associated with greater variability in task performance during the Flanker task. Lower MFG activation was associated with trends toward greater EF impairments, as measured by BRIEF. Taken together, IC challenges seen in autistic youth may be attributed to hypo- or hyperactivation in multiple cortical regions, including DMN and CEN regions, which could serve as potential correlates of EF performance.

### 4.3. Hemispheric Differences Indicate Reduced Lateralization in Autistic Youth

Non-autistic youth had left-lateralized STS activation during the congruent condition and trends toward left-lateralized IPL and MFG activation during the incongruent condition. In contrast, autistic youth exhibited symmetric activation in all ROIs during the congruent conditions and in four out of five ROIs during the incongruent condition. This pattern indicates less left-lateralization and a potential compensatory mechanism of relying on right-hemispheric IPL activation. Autistic individuals are known to exhibit less left-lateralization in language networks, and a more right-lateralized ventral attention network and salient network, as well as a right-lateralized CEN, indicating reduced asymmetry or rightward hemispheric bias during functional activation [60,61]. Such findings were found to be independent of age, sex, and IQ [61]. Similarly, the results of this study did not covary with age and indicated more symmetric functional activation or a rightward bias in IPL activation during IC tasks. Taken together, these exploratory findings may suggest a possible alternative mechanism of rightward bias in cortical activation in autistic youth that may underlie their intact but more variable EF performance.

### 4.4. Regional Differences in Autism Indicate Potential Neural Correlates for IC Challenge

When comparing regional activation within each group, during the congruent (i.e., easier) condition, non-autistic youth showed relatively similar magnitudes of activation within the left hemisphere, with the greatest activation in the STS and IPL regions. However, during the congruent condition, autistic youth showed the lowest activation in the left IPL region, along with a trend toward lower left IPL activation during the incongruent condition. IPL is considered an important part of DMN and CEN regions and is mainly responsible for orienting attention to unexpected stimuli during IC tasks. In contrast, in non-autistic youth, during the congruent condition, left STS activation was the highest, whereas during the incongruent condition, they showed a trend for left STS deactivation (i.e., less activation), along with greater IFG and IPL activation, all of which are crucial regions within the DMN and are considered important for IC [62,63]. Moreover, non-autistic youth exhibited a reciprocal activation pattern between STS and IFG regions across the two Flanker task conditions (congruent and incongruent). However, autistic youth did not exhibit a reciprocal activation pattern between the IFG and STS regions; instead, they showed similar activation in STS and IFG ROIs across both conditions. These findings point to differences in patterns of DMN suppression/deactivation between autistic and non-autistic youth during EF tasks. Overall, fNIRS-based neural correlates in the bilateral IPL, IFG, and STS ROIs could be potential neural correlates of EF performance in autistic youth.

### 4.5. Limitations and Future Directions

This study identified potential neural correlates of IC challenges in school-aged autistic youth using fNIRS-based cortical activation during a naturalistic Flanker task, wherein autistic youth exhibited greater variability in EF performance. There was a correlation between EF performance, as measured through behavioral assessments and standardized questionnaires, and patterns of cortical activation during the Flanker task. These findings hold practical implications, contingent upon further confirmation through subsequent research. First, these outcomes may be utilized as neuro-indicators to explore the efficacy of EF interventions. Furthermore, the results highlight the potential applicability of fNIRS in assessing neural responses to EF interventions and in elucidating the underlying mechanisms of EF in autistic individuals, particularly in contexts where alternative neuroimaging techniques may prove unsuitable. However, it is essential to note that these findings are exploratory, as the current study had a small sample size and a broad age range (5 to 16 years) with varying functional abilities, and IQ data were unavailable. The findings are also task-specific; hence, findings may not simply generalize to EF performance in social settings. In addition, there were no group differences in laterality indices for activation measures, likely due to the small sample size; hence, future studies should further examine differences in lateralization of hemispheric activation in autistic youth. Lastly, the fNIRS spatial registration method is associated with some anatomical uncertainty, which may have influenced our findings. It would be valuable to replicate our findings in larger samples, involving different subgroups based on age, IQ, functioning, and across various EF tasks. Overall, our findings support the use of fNIRS in studying cortical activation differences and their associations with EF performance in autistic youth. Future studies should examine whether fNIRS-based cortical activation differences can serve as neural indicators in assessing intervention response following EF interventions provided to autistic youth.

### 4.6. Conclusions

The current study results indicates that autistic youth exhibited intact but highly variable EF performance during an fNIRS modified Flanker task, along with greater parent-reported EF impairments compared to age-matched non-autistic youth, suggesting delayed EF development and differences in fNIRS-based cortical activation. Specifically, during both conditions, autistic youth showed lower left frontal and parietal region activation compared to non-autistic controls, along with compensatory greater right parietal region activation that correlated with greater reaction time variability in autistic youth, suggestive of EF neural correlates in autistic youth. ANCOVAs followed by the between-group *t*-tests also revealed a lack of left-lateralization (or more symmetric activation or a rightward bias) in autistic youth compared to non-autistic youth, who had more asymmetric activation during EF task performance. Overall, greater variability in EF task performance in autistic youth was associated with hypo- or hyperactivation within frontal–parietal and superior temporal regions, which are important ROIs within the DMN and CEN, making them neural correlates with potential application value. Lastly, these findings underscore the potential utility of fNIRS for assessing intervention responses following EF interventions in autistic youth.

## Figures and Tables

**Table 1:** Demographic information and clinical data for both groups.

Baseline Demographic/Clinical Measure Characteristics	NASD Group (n = 15) Mean ± SE	ASD Group (n = 15) Mean ± SE
Race	4NC, 11C	7NC, 8C
Ethnicity	15NH	13NH, 2H
Sex	9M, 6F	12M, 3F
Handedness	1NRH, 14RH	2NRH, 13RH
Age in Years	11.46 ± 0.85	11.59 ± 0.81
VABS-3, Communication Domain SS *	101.33 ± 2.44	79.4 ± 3.35
VABS-3, Socialization Domain SS *	96.07 ± 2.06	75.27 ± 3.49
VABS-3, Daily Living Domain SS *	95 ± 2.43	84.4 ± 3.69
VABS-3, Motor Domain SS *	99.5 ± 5.35	74.7 ± 2.34
VABS-3, Adaptive Behavior Composite (ABC), SS *	95.71 ± 1.55	76.8 ± 2.56
SRS-2, T-score *	46.5 ± 1.24	69.07 ± 2.28
BRIEF-BRI *	48.27 ± 2.11	69 ± 3.86
BRIEF-MI *	47.6 ± 2.64	70.2 ± 3.35
BRIEF-GEC *	47.8 ± 2.41	71.33 ± 3.69
BOT-2 Body Coordination, SS *	59 ± 2.17	37.07 ± 2.36
BOT-2 Manual Coordination, SS *	47.07 ± 2.07	36.87 ± 0.01

SE = Standard error, M = Male, F = Female; C = Caucasian, NC = Non-Caucasian (Asian, African American, or Biracial), H = Hispanic, NH = Non-Hispanic, RH = Right-Hand Dominant, NRH = Non-Right-Hand Dominant, VABS-3 = Vineland Adaptive Behavior Scale–Third Edition, SS = Standard Score, SRS-2 = Social Responsiveness Scale–Second Edition, GEC = Global Executive Composite, BRI = Behavioral Regulation Index, MI = Meta-Cognition Index, BOT-2 = Bruininks–Oseretsky Test of Motor Proficiency–Second Edition, BRIEF = Behavior Rating Inventory of Executive Function. * near the measure indicates a significant difference between the NASD and ASD groups for the given measure (i.e., *p*-value < 0.05).

## Data Availability

The data presented in this study are available on request from the corresponding author. The data are not publicly available due to privacy and ethical restrictions.

## Supplementary Materials

The following supporting information can be downloaded at: https://www.mdpi.com/article/10.3390/brainsci16010065/s1, Table S1: Channel Assignments following Spatial Registration using the Anchor Registration Approach; Table S2: Percent distribution of activation data (HbO_2_) included per participant and condition. NASD = Non-autistic youth. ASD = Autistic youth; Table S3: Percent distribution of activation data (HbO_2_) included per channel across groups and conditions. NASD = Non-autistic youth. ASD = Autistic youth; Table S4: Mean, Standard Error (SE) of Reaction time and Accuracy per condition and group; Table S5: Group differences in variability of behavioral performance using coefficient of variation (CV) of reaction time. NASD = Non-autistic youth. ASD = Autistic youth; Table S6: Average activation using HbO_2_ values for each Region by Group, Time, Condition, and Hemisphere, during the fNIRS Flanker task. NASD = Non-autistic youth. ASD = Autistic youth; Table S7: Post-hoc comparison data including *p*-values, effect sizes, and direction of effects for the 4-way Group (ASD, NASD) × Hemisphere (left, right) × Condition (congruent, incongruent) x Region (ROIs) interaction effect. NASD = Non-autistic youth. ASD = Autistic youth; Table S8: Correlations between functioning and cortical activation in the ASD group during the fNIRS Flanker task (shown as r, *p* value). * *p* < 0.05; ** *p* < 0.01. Bold font indicates that the *p*-value survived FDR corrections. NA = not applicable. NASD = Non-autistic youth; Table S9: ANOVA statistics on average cortical activation (HbO2) values using the within-group factors of Condition (congruent, incongruent), Hemisphere (left, right), and Region (MFG, IFG, PCG, IPL, STS), and a between-group factor of Group (ASD, NASD). Age was addressed as a covariate. NASD = Non-autistic youth. ASD = Autistic youth; Table S10: ANOVA statistics on behavioral variables (reaction time variability, reaction time, and accuracy) using the within-group factors of Condition (congruent, incongruent), and a between-group factor of Group (ASD, NASD). Age was addressed as a covariate. NASD = Non-autistic youth. ASD = Autistic youth.
