# Supplementary material for: Differences in Executive Functioning Performance and Cortical Activation Between Autistic and Non-Autistic Youth During an fNIRS Flanker Task: A Pilot Study
Source: Brain Sci. 2025 Dec 31;16(1):65. doi: 10.3390/brainsci16010065 (PMC12838736; doi:10.3390/brainsci16010065)
Supplement: Supplementary file 1 [file brainsci-16-00065-s001.zip › brainsci-3936120-supplementary.pdf]

Supplementary Materials

Supplementary Table S1. Channel Assignments following Spatial Registration using the Anchor Registration Approach

		MNI's coordinate system			IFG regions		MFG regions			PCG		IPL regions		STS regions		Region
Hemisphere		X	Y	Z	Inferior frontal gyrus	Lateral orbitofrontal gyrus	Middle orbitofrontal gyrus	Superior frontal gyrus	Middle frontal gyrus	Precentral gyrus	Postcentral gyrus	Supramarginal gyrus	Angular gyrus	Middle temporal gyrus	Superior temporal gyrus	
Left	6	-13.667	54.667	43.333				70.9%	29.1%							MFG
	7	-35.333	40.667	42.333				1.9%	98.1%							MFG
	8	-51	19.667	42.333					95.0%	5.0%						MFG
	9	-60.333	-6.333	43.333						44.7%	55.3%					PCG
	10	-64	-31	46							0.3%	99.7%				IPL
	17	-27.667	57.333	29.333				0.7%	99.3%							MFG
	18	-47.667	38.667	28.667	0.9%				99.1%							MFG
	19	-60	11.667	28.667	23.8%				9.0%	67.2%						PCG
	20	-67	-17	32							41.0%	59.0%				Unclear
	21	-66	-42.667	35.667								88.3%	11.7%			IPL
	27	-16.333	70.333	18				42.2%	57.8%							MFG
	28	-42	55.333	16.333	6.3%				93.7%							MFG
	29	-56.333	30.667	15.667	95.2%				4.8%							IFG
	30	-65.333	-0.333	17.667						52.8%	47.2%					PCG
	31	-69	-29.667	21.667							2.5%	43.5%			54.0%	Unclear
	38	-30	66.667	2	2.6%									97.4%		MFG
	39	-50.667	46.333	2	98.0%	1.7%			0.3%							IFG
	40	-58	16.333	2.667	74.1%					15.8%					10.1%	Unclear
	41	-68.333	-13.667	3.333							11.4%			20.9%	67.7%	STS
	42	-70	-41.333	9.333										36.0%	64.0%	STS
	48	-15.667	69.667	-11			23.3%	52.4%	24.3%							MFG
	49	-41.333	57.667	-10.333	42.7%	47.7%	2.4%		7.2%							IFG
	50	-52.333	32.667	-10.667	24.1%	68.3%									7.6%	IFG
	51	-63	2.333	-11.667										40.4%	59.6%	STS
	52	-71	-25.333	-8.667	0.5%									99.5%		STS
Midline	16	1	63	28.667				R:65.4%, L:33.7%	0.9%							Undefined
	37	2	70	2				R: 53.6%, L:41.1%	5.3%							Undefined
Right	1	67	-28	45								100.0%				IPL
	2	62.667	-1.667	41.667						42.6%	51.9%	5.5%				PCG
	3	52	24.333	41.333	5.5%				90.8%	3.7%						MFG
	4	35.333	44.333	41.333					100.0%							MFG
	5	12	56.667	42.667				61.5%	38.5%							MFG
	11	68	-40.333	35.667								63.3%	36.7%			IPL
	12	69	-12.333	31.333							57.3%	42.7%				Unclear
	13	61	14.667	29.667	17.7%				2.2%	80.1%						PCG
	14	49	40.667	29.667	22.2%				77.8%							MFG
	15	27.667	60	29.667					100.0%							MFG
	22	71	-26.333	21.667							0.7%	49.2%	5.6%		44.5%	Unclear
	23	67.333	3.667	19.333	1.1%					47.7%	51.2%					PCG
	24	58	32.667	18	98.6%					1.4%						IFG
	25	42.667	57	18	26.2%				73.8%							MFG
	26	16.333	71	18.333				14.6%	85.4%							MFG
	32	72	-39.333	8.667										57.8%	42.2%	STS
	33	71	-10.667	3.333							6.7%	86.6%		6.7%		STS
	34	60.667	19.333	5.333	51.0%					38.2%					10.8%	Unclear
	35	52	48.333	2.333	97.6%				2.4%							IFG
	36	31	68	2	3.3%				96.7%							MFG
	43	73	-24.667	-8.667										99.2%	0.8%	STS
	44	65.667	3.333	-11.667										60.2%	39.8%	STS
	45	55	36	-10	57.8%	41.9%									0.3%	IFG
	46	41.333	60	-11.667	40.4%	58.6%			0.9%							IFG
	47	14	71	-12.333			30.0%	29.4%	40.6%							MFG

The spatial location in the MNI coordinate system and the probability of covering different brain regions are shown for each channel. The channels are symmetrically divided across the two hemispheres (left, right). The color-coded channels were considered within a specific ROI - MFG (light blue), IFG (pink), PCG (green), STS (yellow), and IPL (dark blue). Channels were considered “Undefined” (grey in center of the table)” when they covered ROIs across both hemispheres, for example, channels located along the midline. Channels were considered “Unclear” (grey in the last column)” when they substantially covered more than one ROI.

Supplementary Table S2. Percent distribution of activation data (HbO_2) included per participant and condition. NASD = Non-autistic youth. ASD = Autistic youth.

Participant	Congruent (%)	Incongruent (%)	Total (%)
NASD 1	100.00	100.00	100.00
NASD 2	100.00	100.00	100.00
NASD 3	100.00	99.36	99.68
NASD 4	91.99	91.99	91.99
NASD 5	92.95	92.95	92.95
NASD 6	93.27	93.59	93.43
NASD 7	91.99	91.99	91.99
NASD 8	100.00	100.00	100.00
NASD 9	98.08	98.08	98.08
NASD 10	66.35	66.67	66.51
NASD 11	73.72	74.04	73.88
NASD 12	99.36	100.00	99.68
NASD 13	99.68	97.44	98.56
NASD 14	93.59	92.95	93.27
NASD 15	99.68	99.68	99.68
ASD 1	98.40	99.04	98.72
ASD 2	100.00	100.00	100.00
ASD 3	98.72	98.08	98.40
ASD 4	100.00	100.00	100.00
ASD 5	100.00	100.00	100.00
ASD 6	91.99	92.31	92.15
ASD 7	100.00	103.21	101.60
ASD 8	94.55	89.10	91.83
ASD 9	100.00	100.00	100.00
ASD 10	100.00	100.00	100.00
ASD 11	100.00	100.00	100.00
ASD 12	100.00	100.00	100.00
ASD 13	87.82	89.42	88.62
ASD 14	98.08	98.08	98.08
ASD 15	100.00	100.00	100.00

Supplementary Table S3. Percent distribution of activation data (HbO_2) included per channel across groups and conditions. NASD = Non-autistic youth. ASD = Autistic youth.

Channel #	Congruent		Incongruent	
	NASD (%)	ASD (%)	NASD (%)	ASD (%)
1	97.78	95.56	100.00	95.56
2	87.78	93.33	84.44	91.11
3	86.67	95.56	85.56	97.78
4	86.67	100.00	87.78	97.78
5	98.89	98.89	98.89	100.00
6	92.22	100.00	93.33	97.78
7	92.22	92.22	93.33	102.22
8	100.00	98.89	100.00	97.78
9	86.67	93.33	86.67	91.11
10	93.33	93.33	93.33	91.11
11	93.33	96.67	93.33	95.56
12	93.33	100.00	93.33	98.89
13	87.78	95.56	85.56	97.78
14	100.00	100.00	100.00	97.78
15	100.00	100.00	100.00	100.00
16	100.00	100.00	100.00	100.00
17	91.11	87.78	88.89	90.00
18	100.00	92.22	100.00	91.11
19	93.33	100.00	92.22	100.00
20	86.67	92.22	86.67	87.78
21	93.33	100.00	93.33	100.00
22	86.67	100.00	86.67	100.00
23	81.11	100.00	77.78	100.00
24	100.00	93.33	100.00	93.33
25	100.00	100.00	100.00	100.00
26	100.00	100.00	100.00	100.00
27	100.00	100.00	100.00	100.00
28	100.00	100.00	100.00	100.00
29	100.00	100.00	100.00	100.00
30	86.67	97.78	86.67	98.89
31	73.33	93.33	72.22	93.33
32	93.33	100.00	93.33	100.00
33	80.00	100.00	80.00	100.00
34	100.00	100.00	100.00	100.00
35	100.00	100.00	100.00	100.00
36	100.00	100.00	100.00	100.00

37	93.33	100.00	93.33	100.00
38	100.00	100.00	100.00	100.00
39	100.00	100.00	100.00	100.00
40	93.33	100.00	93.33	100.00
41	86.67	94.44	86.67	94.44
42	86.67	90.00	86.67	92.22
43	93.33	100.00	92.22	100.00
44	96.67	100.00	97.78	100.00
45	95.56	100.00	95.56	100.00
46	100.00	100.00	100.00	100.00
47	93.33	100.00	92.22	100.00
48	94.44	100.00	93.33	100.00
49	96.67	100.00	96.67	100.00
50	100.00	100.00	100.00	100.00
51	90.00	100.00	93.33	100.00
52	73.33	100.00	74.44	100.00

Supplementary Table S4. Mean, Standard Error (SE) of Reaction time and Accuracy per condition and group.

	Congruent	SE	Incongruent	SE	Congruent vs. Incongruent (p-values)[a]
Reaction time in milliseconds					
NASD	694.90	26.78	730.62	31.87	0.001*
ASD	681.34	15.89	732.24	18.44	0.022*
Accuracy (%)					
NASD	95.8%	0.005	94.4%	0.007	NS
ASD	95.8%	0.010	95.7%	0.006	NS

NASD = Non-autistic youth. ASD = Autistic youth. NS = Not significant. *t-test significant and survived FDR correction. SE=Standard error. [a]No differences found between or within groups.

Supplementary Table S5. Group differences in variability of behavioral performance using coefficient of variation (CV) of reaction time. NASD = Non-autistic youth. ASD = Autistic youth.

	Overall	Congruent	Incongruent
p-value, Effect size using Hedges' g	0.017, 0.854	0.054, NA	0.004, 1.03
NASD mean	26.37 ± 1.94	24.63 ± 9.41	26.02 ± 1.35
ASD mean	34.8 ± 10.36	34.33 ± 15.90	37.15 ± 3.08

Supplementary Table S6. Average activation using HbO_2 values for each Region by Group, Time, Condition, and Hemisphere, during the fNIRS Flanker task. NASD = Non-autistic youth. ASD = Autistic youth.

**Region**		**Congruent**		**Incongruent**	
		**Mean**	**Standard Error**	**Mean**	**Standard Error**
NASD group					
Right	IPL	0.0016	0.0047	-0.0023	0.0051
	PCG	0.0051	0.0041	-0.0016	0.0057
	MFG	0.0073	0.0032	0.0057	0.0035
	IFG	0.0031	0.0045	0.0081	0.0057
	STS	-0.0018	0.0050	0.00003	0.0050
Left	IPL	0.0088	0.0047	0.0101	0.0056
	PCG	0.0077	0.0039	0.0059	0.0048
	MFG	0.0016	0.0030	0.0059	0.0035
	IFG	0.0044	0.0045	0.0104	0.0051
	STS	0.0170	0.0046	-0.0021	0.0042
ASD group					
Right	IPL	0.0003	0.0084	0.0102	0.0104
	PCG	0.0001	0.0057	-0.0067	0.0048
	MFG	0.0027	0.0033	0.0005	0.0038
	IFG	0.0141	0.0071	0.0133	0.0062
	STS	0.0053	0.0060	0.0083	0.0060
Left	IPL	-0.0109	0.0053	-0.0186	0.0064
	PCG	0.0069	0.0045	-0.0101	0.0060
	MFG	0.0014	0.0036	-0.0043	0.0036
	IFG	0.0105	0.0052	0.0085	0.0055
	STS	0.0127	0.0062	0.0162	0.0060

Supplementary Table S7. Post-hoc comparison data including p-values, effect sizes, and direction of effects for the 4-way Group (ASD, NASD) × Hemisphere (left, right) × Condition (congruent, incongruent) x Region (ROIs) interaction effect. NASD = Non-autistic youth. ASD = Autistic youth.

**Comparisons**	**p-values**	**Direction of effect**	**Effect sizes (Hedges' g)**
Group-Related Differences in Regional Activation			
Left (L) IPL, Congruent	0.006	NASD>ASD	0.415
LIPL, Incongruent	0.0008	NASD>ASD*	0.504
LPCG, Incongruent	0.037	NASD>ASD	0.313
LMFG, Incongruent	0.043	NASD>ASD	0.304
LSTS, Incongruent	0.013	ASD>NASD	0.373
Hemisphere-Related Differences in Regional Activation			
NASD, MFG, Congruent	0.016	R>L	0.197
NASD, STS, Congruent	< 0.001	L>R*	0.412
NASD, IPL, Incongruent	0.04	L>R	0.245
ASD, IPL, Incongruent	0.016	R>L	0.352
Condition-Related Differences in Regional Activation			
NASD, LSTS	0.003	Congruent > Incongruent	0.456
ASD, LPCG	0.025	Congruent > Incongruent	0.338
Region-Related Differences in Regional Activation			
NASD, L Congruent	0.033	STS>IFG	0.291
NASD, L, Congruent	0.006	STS>MFG*	0.417
NASD, L, Incongruent	0.04	IFG>STS	0.282
ASD, L, Congruent	< 0.001	PCG>IPL*	0.381
ASD, L, Congruent	0.041	MFG>IPL	0.286
ASD, L, Congruent	0.004	IFG>IPL*	0.429
ASD, L, Congruent	0.001	STS>IPL*	0.433
ASD, Right (R), Incongruent	0.033	IFG>MFG	0.264
ASD, R, Incongruent	0.003	IFG>PCG*	0.382
ASD, R, Incongruent	0.006	STS>PCG*	0.292
ASD, L, Incongruent	0.004	IFG>MFG*	0.288
ASD, L, Incongruent	0.031	MFG>IPL	0.291
ASD, L, Incongruent	0.018	IFG>PCG	0.34
ASD, L, Incongruent	0.004	STS>MFG*	0.435
ASD, L, Incongruent	0.002	IFG>IPL*	0.477
ASD, L, Incongruent	< 0.001	STS>PCG*	0.462
ASD, L, Incongruent	< 0.001	STS>IPL*	0.591

* p-value survived FDR-correction. Only the comparisons with a p-value of less than 0.05 are provided.

Supplementary Table S8. Correlations between functioning and cortical activation in the ASD group during the fNIRS Flanker task (shown as r, p value). *p < 0.05; **p < 0.01. Bold font indicates that the p-value survived FDR corrections. NA = not applicable. NASD = Non-autistic youth.

**Congruent**						
Right						
	ROIs	IPL	PCG	MFG	IFG	STS
fNIRS Flanker task	Congruent Reaction Time Variability	-0.237*, 0.025	**-0.369**, <0.001**	-0.075, 0.484	-0.132, 0.216	-0.221*, 0.037
	Incongruent Reaction Time Variability	NA	NA	NA	NA	NA
	Congruent Reaction Time	**0.385**, <.001**	0.031, 0.771	0.004, 0.97	**0.292**, 0.005**	-0.015, 0.886
	Incongruent Reaction Time	NA	NA	NA	NA	NA
VABS-3 Standard Scores	Communication	0.136, 0.20	0.156, 0.142	-0.038, 0.722	0.016, 0.881	0.07, 0.514
	Socialization	0.107, 0.317	-0.008, 0.938	-0.043, 0.686	0.027, 0.80	-0.009, 0.933
	Daily Living	0.345**, <.001	-0.037, 0.726	-0.013, 0.905	0.169, 0.112	0.014, 0.899
	Overall Adaptive Functioning	0.220*, 0.037	0.036, 0.737	-0.043, 0.687	0.062, 0.56	0.023, 0.833
SRS-2 T Scores	Social Responsiveness	**-0.302**, 0.004**	-0.249*, 0.018	-0.244*, 0.02	-0.264*, 0.012	-0.127, 0.232
RBSR total score	Repetitive Behaviors severity	-0.123, 0.249	-0.002, 0.982	-0.058, 0.587	0.024, 0.821	0.021, 0.846
BRIEF Index Scores	Behavioral Regulation	-0.182, 0.085	-0.229*, 0.03	-0.261*, 0.013	-0.260*, 0.013	-0.128, 0.23
	Meta-cognition	**-.0340**, 0.001**	-0.106, 0.319	-0.176, 0.097	**-.0310**, 0.003**	0.015, 0.892
	Global Executive Composite	-.287**, 0.006	-0.168, 0.113	-.227*, 0.031	**-.0305**, 0.004**	-0.047, 0.661
BOT-2 T scores	Manual Coordination	.264*, 0.012	0.075, 0.484	0.195, 0.065	.218*, 0.039	0.134, 0.207
	Age	**-.370**, <.001**	-0.075, 0.482	-0.079, 0.459	-.259*, 0.014	-0.007, 0.95
**Congruent**						
Left						
	ROIs	IPL	PCG	MFG	IFG	STS
fNIRS Flanker task	Congruent Reaction Time Variability	-0.236*, 0.025	-0.111, 0.299	-0.071, 0.503	0.137, 0.199	-0.092, 0.389
	Incongruent Reaction Time Variability	NA	NA	NA	NA	NA
	Congruent Reaction Time	-0.143, 0.179	0.031, 0.773	0.091, 0.395	0.086, 0.421	**-0.284**, 0.007**
	Incongruent Reaction Time	NA	NA	NA	NA	NA
VABS-3 Standard Scores	Communication	0.173, 0.103	0.058, 0.589	0.042, 0.692	-0.202, 0.056	-0.158, 0.137
	Socialization	0.134, 0.208	0.105, 0.324	0.09, 0.398	-0.127, 0.234	-0.206, 0.051
	Daily Living	0.116, 0.278	0.144, 0.177	0.153, 0.151	-0.004, 0.968	-0.330**, 0.001
	Overall Adaptive Functioning	0.17, 0.109	0.104, 0.331	0.094, 0.377	-0.123, 0.248	-0.250*, 0.017
SRS-2 T Scores	Social Responsiveness	0.079, 0.46	-0.097, 0.365	-.258*, 0.014	0.089, 0.402	0.109, 0.308
RBSR total score	Repetitive Behaviors severity	-0.176, 0.098	-0.053, 0.621	-0.072, 0.498	-0.074, 0.485	0.105, 0.326
BRIEF Index Scores	Behavioral Regulation	0.006, 0.958	-0.136, 0.20	-0.18, 0.089	-0.009, 0.934	-0.109, 0.306
	Meta-cognition	-0.011, 0.918	-0.104, 0.327	-0.248*, 0.019	-0.051, 0.632	0.086, 0.423
	Global Executive Composite	0.004, 0.973	-0.118, 0.269	-0.240*, 0.022	-0.035, 0.743	0.014, 0.897
BOT-2 T scores	Manual Coordination	-0.024, 0.819	0.105, 0.325	0.085, 0.426	-0.005, 0.963	0.012, 0.911
	Age	0.174, 0.101	-0.005, 0.962	-0.082, 0.44	0.143, 0.179	0.157, 0.139
**Incongruent**						
Right						
	ROIs	IPL	PCG	MFG	IFG	STS
fNIRS Flanker task	Congruent Reaction Time Variability	NA	NA	NA	NA	NA
	Incongruent Reaction Time Variability	-.218*, 0.039	-0.104, 0.328	0.08, 0.454	0.027, 0.798	-.239*, 0.023
	Congruent Reaction Time	NA	NA	NA	NA	NA
	Incongruent Reaction Time	-0.025, 0.817	**0.382**, <.001**	0.094, 0.379	-0.021, 0.848	0.192, 0.07
VABS-3 Standard Scores	Communication	0.155, 0.146	0.051, 0.632	-0.053, 0.62	-0.14, 0.187	0.002, 0.988
	Socialization	-0.026, 0.805	-0.116, 0.276	-0.083, 0.436	-0.118, 0.267	-0.152, 0.154
	Daily Living	0.141, 0.185	-0.031, 0.77	0.094, 0.379	-0.014, 0.895	-0.14, 0.187
	Overall Adaptive Functioning	0.085, 0.426	-0.033, 0.758	-0.044, 0.678	-0.12, 0.259	-0.14, 0.188
SRS-2 T Scores	Social Responsiveness	-0.134, 0.207	0.028, 0.795	**-0.306**, 0.003**	0.029, 0.789	0.006, 0.952
RBSR total score	Repetitive Behaviors severity	0.014, 0.893	0.011, 0.918	0.014, 0.897	0.090, 0.40	0.041, 0.698

BRIEF Index Scores	Behavioral Regulation	0.03, 0.778	0.094, 0.379	-0.189, 0.074	-0.006, 0.954	0.04, 0.708
	Meta-cognition	-0.129, 0.227	0.136, 0.201	-0.195, 0.066	-0.021, 0.841	0.086, 0.421
	Global Executive Composite	-0.069, 0.516	0.113, 0.29	-.215*, 0.042	-0.021, 0.844	0.065, 0.541
BOT-2 T scores	Manual Coordination	0.06, 0.573	-0.037, 0.731	0.193, 0.069	0.126, 0.237	-0.079, 0.462
	Age	**-0.411**, <0.001**	0.03, 0.778	**-0.294**, 0.005**	-0.094, 0.38	-0.116, 0.278
**Incongruent**						
Left						
	ROIs	IPL	PCG	MFG	IFG	STS
fNIRS Flanker task	Congruent Reaction Time Variability	NA	NA	NA	NA	NA
	Incongruent Reaction Time Variability	-0.114, 0.283	-0.163, 0.125	0, 0.998	0.125, 0.24	-0.057, 0.591
	Congruent Reaction Time	NA	NA	NA	NA	NA
	Incongruent Reaction Time	0.266*, 0.011	0.041, 0.701	-0.009, 0.93	0.018, 0.867	0.199, 0.06
VABS-3 Standard Scores	Communication	0.228*, 0.031	0.209*, 0.048	-0.038, 0.726	-.212*, 0.045	0.04, 0.705
	Socialization	0.163, 0.125	0.056, 0.60	-0.011, 0.916	-0.170, 0.11	-0.105, 0.323
	Daily Living	0.155, 0.144	0.069, 0.521	0.159, 0.135	-0.038, 0.722	-0.205, 0.052
	Overall Adaptive Functioning	0.207, 0.05	0.124, 0.243	0.021, 0.846	-0.186, 0.079	-0.123, 0.247
SRS-2 T Scores	Social Responsiveness	-0.114, 0.287	0.244*, 0.021	-0.046, 0.669	0.073, 0.494	0.156, 0.142
RBSR total score	Repetitive Behaviors severity	-0.132, 0.216	0.096, 0.367	0.041, 0.703	0.061, 0.571	-0.084, 0.43
BRIEF Index Scores	Behavioral Regulation	-0.074, 0.49	**0.360**, <0.001**	0.041, 0.703	0.099, 0.352	0.195, 0.065
	Meta-cognition	-0.026, 0.807	**0.354**, <0.001**	-0.022, 0.836	0.035, 0.741	0.201, 0.058
	Global Executive Composite	-0.048, 0.656	**0.375**, <0.001**	-0.007, 0.947	0.052, 0.626	0.204, 0.054
BOT-2 T scores	Manual Coordination	0.071, 0.508	-0.065, 0.542	0.083, 0.434	-0.048, 0.656	-0.156, 0.142
	Age	0.049, 0.65	-0.025, 0.812	-0.084, 0.43	0.02, 0.854	0.083, 0.439

Supplementary Table S9. ANOVA statistics on average cortical activation (HbO2) values using the within-group factors of Condition (congruent, incongruent), Hemisphere (left, right), and Region (MFG, IFG, PCG, IPL, STS), and a between-group factor of Group (ASD, NASD). Age was addressed as a covariate. NASD = Non-autistic youth. ASD = Autistic youth.

Effect/ Error	df	Mean Square	F	p-value	η_p^2
Age	1	0.019	1.857	0.175	0.010
Group	1	0.003	0.246	0.621	0.001
Error	177	0.010	NA	NA	NA
Condition	1	0.001	0.202	0.654	0.001
Condition x Group	1	0.000	0.043	0.836	0.000
Error (Condition)	177	0.006	NA	NA	NA
Hemisphere	1	0.020	7.895	0.006	0.043
Hemisphere x Group	1	0.015	6.030	0.015	0.033
Error (Hemisphere)	177	0.002	NA	NA	NA
Region	3	0.012	3.014	0.029	0.017
Region x Group	3	0.013	3.382	0.017	0.019
Error (Region)	540	0.004	NA	NA	NA
Condition x Hemisphere	1	0.000	0.106	0.745	0.001
Condition x Hemisphere x Group	1	0.002	0.995	0.320	0.006
Error (Condition x Hemisphere)	177	0.002	NA	NA	NA
Condition x Region	3	0.001	0.525	0.685	0.003
Condition x Region x Group	3	0.004	1.791	0.141	0.010
Error (Condition x Region)	593	0.002	NA	NA	NA
Hemisphere x Region	3	0.009	4.292	0.005	0.024
Hemisphere x Region x Group	3	0.009	4.745	0.003	0.026
Error (Hemisphere x Region)	528	0.002	NA	NA	NA
Condition x Hemisphere x Region	3	0.000	0.259	0.866	0.001
Condition x Hemisphere x Region x Group	3	0.004	2.633	0.046	0.015
Error (Condition x Hemisphere x Region)	565	0.002			

Supplementary Table S10. ANOVA statistics on behavioral variables (reaction time variability, reaction time, and accuracy) using the within-group factors of Condition (congruent, incongruent), and a between-group factor of Group (ASD, NASD). Age was addressed as a covariate. NASD = Non-autistic youth. ASD = Autistic youth.

Effect/ Error	df	Mean Square	F	p-value	η_p^2
**Reaction Time Variability**					
Age	1	76.768	0.400	0.528	0.002
Group	1	9782.662	51.016	0.000	0.224
Error	177	191.758	NA	NA	NA
condition	1	2.454	0.049	0.825	0.000
condition x group	1	44.992	0.898	0.345	0.005
Error (condition)	177	50.090	NA	NA	NA
**Mean Reaction Time**					
Age	1	8751898.225	191.781	0.000	0.520
Group	1	695.561	0.015	0.902	0.000
Error	177	45634.953	NA	NA	NA
Condition	1	65856.472	6.581	0.011	0.036
Condition x group	1	5411.932	0.541	0.463	0.003
Error (Condition)	177	10006.680	NA	NA	NA
**Mean Accuracy**					
Age	1	8751898.225	191.781	0.000	0.520
Group	1	695.561	0.015	0.902	0.000
Error	177	45634.953	NA	NA	NA
Condition	1	0.006	1.943	0.165	0.011
Condition x group	1	0.004	1.061	0.304	0.006
Error (Condition)	177	0.003	NA	NA	NA
